# A Severe Diabetic Foot Ulcer With Intermediate Cuneiform Displacement and Multidrug-Resistant *Pseudomonas aeruginosa* Infection: A Rare Case Report

**DOI:** 10.3389/fmed.2020.00131

**Published:** 2020-04-21

**Authors:** Wei Chai, Yuqing Wang, Fengjuan Jiao, Yili Wu, Shuai Wang

**Affiliations:** ^1^Department of Surgery, Tianjin Second Hospital, Tianjin, China; ^2^Cheeloo College of Medicine, Shandong University, Jinan, China; ^3^Shandong Key Laboratory of Behavioral Medicine, School of Mental Health, Jining Medical University, Jining, China; ^4^Shandong Collaborative Innovation Center for Diagnosis, Treatment & Behavioral Interventions of Mental Disorders, Institute of Mental Health, Jining Medical University, Jining, China

**Keywords:** diabetic foot ulcer, intermediate cuneiform displacement, multidrug-resistant infection, debridement, wound dressing, antibiotic selection

## Abstract

Diabetic foot ulcer (DFU) is considered as one of the most serious and prevailing complications of diabetes mellitus, while it is the major cause of amputations in diabetic patients. Herein, we reported an acquired severe traumatic DFU with an intermediate cuneiform hairline fracture and displacement in a 55-year old male (Grade IV of Wagner classification; Grade III of IWGDF classification). The *Pseudomonas aeruginosa* was identified in pus culture. Data of antibiotic susceptibility testing indicated that the isolates of *Pseudomonas aeruginosa* were multi-drug resistant. Routine debridement, clearing displaced intermediate cuneiform and drainage were performed to facilitate the outflow of pus and pressure mitigation. Dressing with Prontosan solution and gel was applied to the wound, and meropenem was systemically administrated in addition to effective glycemic control. The DFU has been fully healed after ~40-day treatment. For this case, clearing the displaced and fractured intermediate cuneiform is essential for the heal of the DFU in addition to the common strategy for DFU treatment, i.e., the combination of debridement, pressure mitigation, wound dressing with Prontosan, antibiotic selection and effective glycemic control. This case report might have value for the treatment of complex DFU with bone fracture and displacement, reducing the risk of amputation.

## Background

Metabolic disfunction in diabetes mellitus (DM) can lead to secondary inevitable pathophysiological outcomes on multiple organ systems (1). Diabetic foot (DF), one of the common complications of DM, often presents with neuropathy and minor vascular lesions in the lower limbs. Due to various risk factors such as peripheral neuropathy, peripheral vascular disease, foot deformities, and arterial insufficiency (2), diabetic patients are prone to develop diabetic foot ulcer (DFU) (3), accompanied by local tissue necrosis and easily leading to gangrene and ineluctable amputation in extreme cases with infection (4, 5). Ulceration and tissues destruction accompanied by infection seriously impair patients' quality of life. According to IDSA (Infectious Diseases Society of America) classification, around 46 and 70% of patients with moderate and severe diabetic foot infection require an amputation, respectively (6). In this report, we presented a healing case of severe DFU with an intermediate cuneiform hairline fracture and displacement. The DFU has been fully healed without amputation by clearing the displaced and fractured intermediate cuneiform, along with the combination of debridement, pressure mitigation, wound dressing with Prontosan, antimicrobials, and effective glycemic control.

## Case Presentation

A 55-year-old man was admitted to our department for swelling, pain, and deteriorative ulcer on his left-foot dorsum. The patients had a diabetes history for over 12 years. Although oral repaglinide (2 mg, three times a day) and metformin (0.5 g, three times a day) were taken before ulceration, the patient had a long-term poorly controlled glycaemia. Loss of consciousness resulted in the patient falling at home 20 days before hospitalization. After feeding, he regained consciousness gradually without other medical treatment. However, 15 days before hospitalization, swelling with pain presented on left-foot dorsum, and an ulcer grew on the raised surface of the swelling with progressive deteriorative phenotype 10 days later. After non-effective self-treatment, the patient was admitted to our department.

**Figure 1:**
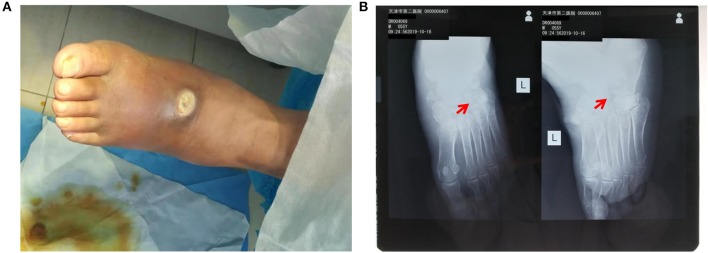
Pre-operative detection showed swelling and intermediate cuneiform displacement in the left foot. **(A)** Distinct swelling and 2 × 3-cm sized ulcer presented on the surface of left-foot dorsum; **(B)** X-ray detection revealed intermediate cuneiform fracture and displacement. The red arrows indicate the position of displaced intermediate cuneiform.

Physical examination showed a swelling and a 2 × 3-cm sized ulcer on the left-foot dorsum. Apparent ischemic black margin and potential infection induced gangrene was observed (Figure 1A). A small amount of yellow pus could overflow from the ulcer under slight pressure. General test showed the patient with body temperature of 38.8°C blood pressure of 110/60 mmhg, and heart rate of 96 min^−1^. Neurologic physical examination showed weakened algesia, pselaphesia, thermesthesia, and vibration sense in the left lower limb. X-ray detection revealed the hairline fracture and instep-orientated displacement of intermediate cuneiform (Figure 1B). Electromyography examination showed delayed onset of action potential in the left lower limb indicating peripheral neuropathy. Color Doppler detection showed multiple mural plaques in the left superficial femoral artery with the estimated stenosis of 50%. Blood routine showed a moderate anemia with the lower erythrocytes count of 2.64^*^10^∧^12/L and lower hemoglobin concentration of 83 g/L. The fasting and post-prandial blood glucose levels were 234 mg/dl (13 mmol/L) and 360 mg/dl (20 mmol/L), respectively. The HbA1c level was 13.1% (Table 1). Based on these evidence, the patient preliminary diagnosed as diabetic foot ulcer (Grade IV of Wagner classification; Grade III of IWGDF classification) with infection, fracture, and displacement of intermediate cuneiform, and moderate anemia.

**Table 1:** Details of blood routine and blood glucose level.

**Indices**	**Pre-therapy**	**Post-therapy**	**Normal range**
Leukocyte count	5.78*10^∧^9/L	4.06*10^∧^9/L	(4.0–10.0)*10^∧^9/L
Erythrocytes count	2.64*10^∧^12/L	3.23*10^∧^12/L	(3.5–5.5)*10^∧^12/L
Hemoglobin concentration	83 g/L	99 g/L	110–150 g/L
Platelet count	552*10^∧^9/L	398*10^∧^9/L	(100–300)*10^∧^9/L
Fasting blood glucose level	234 mg/dl	127.8 mg/dl	70.2–109.8 mg/dl
Post-prandial blood glucose level	360 mg/dl	180 mg/dl	126–180 mg/dl
Glycosylated hemoglobin A1c	13.1%	6.8%	3.9–6.1%

**Figure 2:**
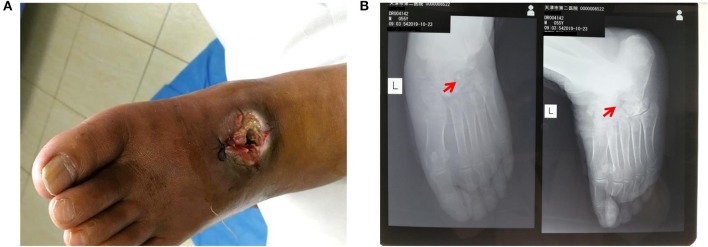
Post-operative detection showed successful debridement and ebonation. **(A)** Alleviated swelling presented on the surface of left-foot dorsum after debridement; **(B)** X-ray film view showed that the displaced intermediate cuneiform was removed. The red arrows indicate the position of removed intermediate cuneiform.

**Figure 3:**
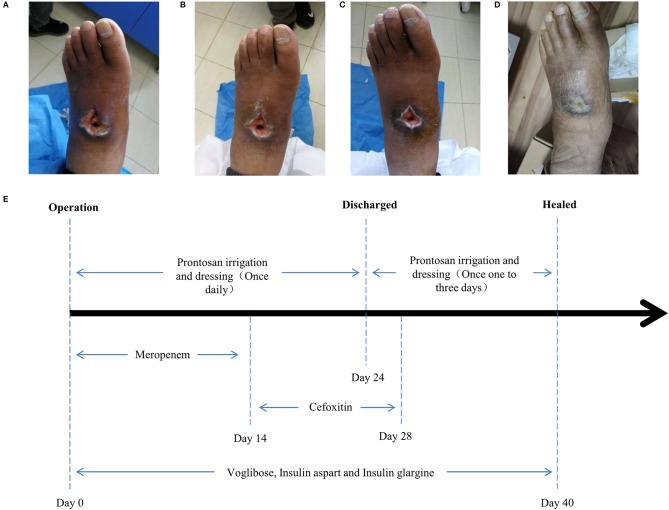
Wound conditions at the **(A)** 7th, **(B)** 14th, **(C)** 24th, and **(D)** 40th day of posttherapy showed the gradual healing of ulceration and the timeline **(E)** of the treatment process from the day of operation (day 0) to the day of healing (day 40).

Surgery was firstly performed to accelerate outflow of pus inside the ulcer in order to reduce pressure inside the swelling. Surgical incision was traced along proximal and distal end of wound surface, revealing the displaced intermediate cuneiform below, which obstructed the drainage operation. Clearing the obstruction by removing the displaced intermediate cuneiform (Figures 2A,B), ~50 ml yellow pus gushed from the wound. A 4 cm deep vomica close to arch was uncovered during surgery. Highly resistant strain of *Pseudomonas aeruginosa* was detected. Drug sensitivity test showed chloramphenicol, rifampicin, and vancomycin were the most effective antibiotics on *P. aeruginosa* inhibition, with the minimal inhibit concentration (MIC) of <1, ≤ 1, and <0.5, respectively. However, considering the severe side effects of these three antibiotics (hepatic and renal impairment), meropenem, the moderately effective antibiotics with MIC of 4, was selected. Intravenous drip of meropenem (2 g/day, twice a day) was administrated for 14 days. Subsequently, it was replaced with oral cefoxitin (0.5 g/day, four times a day), another moderately effective antibiotics, for 14 days. Meanwhile, effective glycemic control was achieved by giving the following medications, subcutaneous injection of insulin aspart (6 IU, three times a day), a rapid-acting form of insulin, during each meal, oral voglibose tablet (0.4 mg, three times a day), an inhibitor of α- glycosidase impeding the hydrolysis of disaccharides into monosaccharides, after each meal and subcutaneous injection of insulin glargine (17 IU, once per night), a long-acting form of insulin. The fasting and post-prandial blood glucose levels were reduced to 127.8 mg/dl (7.1 mmol/L) and 180 mg/dl (10 mmol/L), respectively. The HbA1c level was reduced to 6.8% (Table 1). Importantly, Prontosan solution containing 0.1% undecylenamidopropyl betaine and 0.1% polihexanide was used to treat wound infection locally. Briefly, Prontosan solution irrigation, external dressings with Prontosan solution and Prontosan gel have been applied as local anti-infection treatment for 40 days from the right postoperative day. A week after surgery, pus was still overflowing from the ulcer wound (Figure 3A). Two weeks later, clear and fresh surface of ulcer presented while necrotic tissue also existed (Figure 3B). Twenty-four days after operation, the size of ulcer was distinctly shrunk and granulation tissue but not necrotic tissue was apparent on the surface (Figure 3C), thus the patient was discharged. Then, irrigation and dressing change were applied every 1–3 days until the ulcer healed entirely, ~40 days after operation (Figure 3D). Importantly, the patient was proposed to avoid strenuous exercise such as running and jumping after recovery although there was no evident effect on walking. To be clear, the timeline of entire treatment process was presented in Figure 3E.

## Discussion

DFU is considered as one of the most serious and prevailing complications of DM, which imposes a huge burden on health care system (7). Diabetic peripheral neuropathy is a common risk factor for DFU. Neuropathy related alterations in motor, sensation, and autonomic dysfunctions facilitate accidental injury without protective sensibility.

Once the protective layer of skin is broken and a wound is formed, deep tissues are exposed to pathogens (8). Infection is accelerated by DM-related immunological deficits and rapidly progresses into the deep tissues. Therefore, infection is a consequence rather than a cause of DFU. All infections initiated as a slight problem may progress into deep tissues, joints, or bones especially if not managed, leading to amputation in some extreme cases (9). In this case, the fracture and displacement of intermediate cuneiform caused by accidental fall damaged deep tissues of left foot but was ignored due to impaired sensibility resulted from DM-associated neuropathy until ulceration and infection occurred. X-ray detection confirmed the intermediate cuneiform fracture and displacement, which might cause impediment of pus outflow, deteriorating local hematoma and fester formation. Therefore, surgical drainage was performed after obstruction clearing, which was beneficial for pus outflow.

Wound infection is a common and serious problem in patients with diabetes, and is admitted as a risk factor for lower extremity amputations, especially with multidrug-resistant infection (10–12). On the surface of ulceration, structured community of microbial cells would form a biofilm gradually to advance their own survival. Biofilms is likely to impede wound healing and reduce effectiveness of antibiotics or other antimicrobial agents (13, 14). According to 2019 International Working Group on the Diabetic Foot (IWGDF) Guidelines on the prevention and management of diabetic foot disease (https://iwgdfguidelines.org/guidelines/guidelines/) (15), pathogen detection and drug sensitivity tests are performed to narrow antibiotics spectrum in order to avoid antibacterial resistance and other adverse outcome of the therapy. In this case, pathogen detection and drug sensitivity tests revealed that the major pathogen was multidrug-resistant *Pseudomonas aeruginosa*. As the most common gram-negative bacteria isolated from DF infection (16.9%) (16), *P aeruginosa* can heavily delay DFU healing (7). As infection is difficult to be controlled in such cases, amputation is one of the most common consequences. For this patient, Prontosan dressing with the active ingredient of undecylenamidopropyl betaine and polihexanide was applied to create a moist environment on the surface of the ulceration in order to destroy the biofilm, further promoting granulation, autolytic processes, angiogenesis, and migration of epidermal cells across the wound base (6, 17, 18). Prontosan dressing and systematical application of antibiotics (meropenem or cefoxitin) synergistically inhibited deterioration of infection and facilitated wound healing. Additionally, glycemic control is also crucial for DFU treatment. Plenty of evidence from randomized clinical trials demonstrates that both hyperglycemia and hypoglycemia are associated with prolonged hospital stay and poor clinical outcomes including amputation (19, 20). In this case, insulin therapy combined with voglibose tablet worked effectively on the remission of hyperglycemia, and proper glycemic control after surgery was essential for infection inhibition and wound healing. For this patient, the severe DFU were mainly caused by delayed treatment in addition to accidental trauma and longtime poor glycemic control. Therefore, the patient was advised to keep effective glycemic control. Moreover, once trauma or spontaneous ulceration appeared, timely treatment was necessary. Although no evident effect on walking after recovery was observed, the patient was proposed to avoid strenuous exercise such as running and jumping. In conclusion, clearing the displaced and fractured intermediate cuneiform is essential for the healing of the DFU in addition to the common strategy for DFU treatment. This case report might have value for the treatment of complex DFU with bone fracture and displacement, reducing the risk of amputation.

## Data Availability Statement

All datasets generated for this study are included in the article/supplementary material.

## Ethics Statement

The studies involving human participants were reviewed and approved by Ethics committee of Jining Medical University. The patients/participants provided their written informed consent to participate in this study.

## Author Contributions

WC, SW, and YWu: conception and design of the work. WC: data collection. WC, YWa, FJ, YWu, and SW: data analysis and interpretation, manuscript writing, and critical revision of the article. WC, YWa, FJ, YWu, and SW: approval of the final version of the article.

## Conflict of Interest

The authors declare that the research was conducted in the absence of any commercial or financial relationships that could be construed as a potential conflict of interest.

## References

[1] Lim JZ Ng NS Thomas C. Prevention and treatment of diabetic foot ulcers. J R Soc Med. (2017) 110:104–9. 10.1177/0141076816688346 28116957 PMC5349377

[2] Noor S Zubair M Ahmad J. Diabetic foot ulcer–a review on pathophysiology, classification and microbial etiology. Diabetes Metab Syndr. (2015) 9:192–9. 10.1016/j.dsx.2015.04.007 25982677

[3] Khanolkar MP Bain SC Stephens JW. The diabetic foot. QJM. (2008) 101:685–95. 10.1093/qjmed/hcn027 18353793

[4] Bakker K Apelqvist J Lipsky BA Van Netten JJ International Working Group on the Diabetic F. The 2015 IWGDF guidance documents on prevention and management of foot problems in diabetes: development of an evidence-based global consensus. Diabetes Metab Res Rev. (2016) 32(Suppl 1):2–6. 10.1002/dmrr.2694 26409930

[5] Bakker K Schaper NC International Working Group on Diabetic Foot Editorial B. The development of global consensus guidelines on the management and prevention of the diabetic foot 2011. Diabetes Metab Res Rev. (2012) 28(Suppl 1):116–8. 10.1002/dmrr.2254 22271736

[6] Lipsky BA Berendt AR Cornia PB Pile JC Peters EJ Armstrong DG 2012 Infectious Diseases Society of America clinical practice guideline for the diagnosis and treatment of diabetic foot infections. Clin Infect Dis. (2012) 54:e132–73. 10.1093/cid/cis346 22619242

[7] Weigelt JA. Diabetic foot infections: diagnosis and management. Surg Infect. (2010) 11:295–8. 10.1089/sur.2010.028 20553107

[8] Johani K Malone M Jensen S Gosbell I Dickson H Hu H. Microscopy visualisation confirms multi-species biofilms are ubiquitous in diabetic foot ulcers. Int Wound J. (2017) 14:1160–9. 10.1111/iwj.12777 28643380 PMC7949972

[9] Vileikyte L Pouwer F Gonzalez JS. Psychosocial research in the diabetic foot: are we making progress? Diabetes Metab Res Rev. (2019) 36:e3257. 10.1002/dmrr.3257 31850665

[10] Acar E Kacira BK. Predictors of lower extremity amputation and reamputation associated with the diabetic foot. J Foot Ankle Surg. (2017) 56:1218–22. 10.1053/j.jfas.2017.06.004 28765052

[11] Ferreira L Carvalho A Carvalho R. Short-term predictors of amputation in patients with diabetic foot ulcers. Diabetes Metab Syndr. (2018) 12:875–9. 10.1016/j.dsx.2018.05.007 29802073

[12] Sen P Demirdal T Emir B. Meta-analysis of risk factors for amputation in diabetic foot infections. Diabetes Metab Res Rev. (2019) 35:e3165. 10.1002/dmrr.3165 30953392

[13] Snyder RJ Bohn G Hanft J Harkless L Kim P Lavery L. Wound biofilm: current perspectives and strategies on biofilm disruption and treatments. Wounds. (2017) 29:S1–17. 28682297

[14] Rahim K Saleha S Zhu X Huo L Basit A Franco OL. Bacterial contribution in chronicity of wounds. Microb Ecol. (2017) 73:710–21. 10.1007/s00248-016-0867-9 27742997

[15] Hinchliffe RJ Forsythe RO Apelqvist J Boyko EJ Fitridge R Hong JP. Guidelines on diagnosis, prognosis, and management of peripheral artery disease in patients with foot ulcers and diabetes (IWGDF 2019 update). Diabetes Metab Res Rev. (2020) 36:e3276. 10.1002/dmrr.3276 31958217

[16] Ramakant P Verma AK Misra R Prasad KN Chand G Mishra A. Changing microbiological profile of pathogenic bacteria in diabetic foot infections: time for a rethink on which empirical therapy to choose? Diabetologia. (2011) 54:58–64. 10.1007/s00125-010-1893-7 20835702

[17] Braun L Kim PJ Margolis D Peters EJ Lavery LA Wound Healing S. What's new in the literature: an update of new research since the original WHS diabetic foot ulcer guidelines in 2006. Wound Repair Regen. (2014) 22:594–604. 10.1111/wrr.12220 25139424

[18] Bakker K Apelqvist J Schaper NC International Working Group on Diabetic Foot Editorial B. Practical guidelines on the management and prevention of the diabetic foot 2011. Diabetes Metab Res Rev. (2012) 28(Suppl 1):225–31. 10.1002/dmrr.2253 22271742

[19] Adler AI Erqou S Lima TA Robinson AH. Association between glycated haemoglobin and the risk of lower extremity amputation in patients with diabetes mellitus-review and meta-analysis. Diabetologia. (2010) 53:840–9. 10.1007/s00125-009-1638-7 20127309

[20] Peled S Pollack R Elishoov O Haze A Cahn A. Association of inpatient glucose measurements with amputations in patients hospitalized with acute diabetic foot. J Clin Endocrinol Metab. (2019) 104:5445–52. 10.1210/jc.2019-00774 31246256

